# Identifying the Most Sensitive COP Variable in Static Balance: The Impact of Chronic Ankle Instability on Postural Stability

**DOI:** 10.1002/jfa2.70072

**Published:** 2025-08-01

**Authors:** Mahdi Majlesi

**Affiliations:** ^1^ Department of Sport Biomechanics Ha.C. Islamic Azad University Hamedan Iran

**Keywords:** balance assessment, center of pressure, chronic ankle instability, postural stability

## Abstract

**Background:**

Chronic ankle instability (CAI) is associated with deficits in postural control, commonly evaluated using center of pressure (COP) variables. Identifying the most sensitive COP measures for detecting balance impairments in individuals with CAI is essential for enhancing assessment and rehabilitation strategies. This study aimed to determine the most sensitive COP variables for evaluating postural stability in individuals with CAI compared to healthy controls.

**Methods:**

Forty participants (20 with CAI, 20 healthy controls) performed single‐leg stance tasks on a force plate. COP variables—including average displacement, standard deviation, root mean square (RMS), range, slope, acceleration, velocity, and integral—were analyzed in anteroposterior (AP) and mediolateral (ML) directions. Repeated measures ANOVA was used for statistical comparisons.

**Results:**

For the injured limb, ML RMS was significantly higher in the CAI group (*p* = 0.049), whereas AP variables showed no significant differences. In the noninjured limb, ML velocity was higher in controls (*p* = 0.049), whereas the integral value was greater in the CAI group (*p* = 0.042). Significant foot*direction interactions were observed for acceleration, velocity (*p* < 0.001), and integral (*p* = 0.045).

**Conclusions:**

ML RMS and integral were the most sensitive COP variables for detecting postural stability deficits in CAI individuals. These findings underscore the importance of ML‐specific balance assessments and targeted rehabilitation strategies to enhance postural control in this population.

## 1 Introduction

Ankle sprains are one of the most common musculoskeletal injuries, frequently occurring during sports that involve rapid changes in direction, jumping, or landing, such as basketball, soccer, and volleyball [1]. These injuries often lead to chronic ankle instability (CAI), which is associated with impaired postural control and an increased risk of recurrent sprains [2]. Postural stability is a fundamental component of human movement, essential for both daily activities and athletic performance [3]. One of the key systems involved in postural control is the sensorimotor system, particularly proprioception, which is often disrupted following musculoskeletal injuries such as ankle sprains, leading to balance deficits [4].

Central to postural control is the concept of the center of pressure (COP), a critical variable for assessing balance and stability. COP reflects the point of application of the ground reaction force vector and is widely used in research to evaluate postural control mechanisms [5]. By analyzing COP displacement characteristics—such as sway area, velocity, and path length—researchers can assess balance performance and identify deficits associated with CAI. Given the role of proprioception in maintaining balance, investigating COP variables can provide valuable insights into the impact of ankle injuries on postural stability and help develop targeted rehabilitation strategies.

Chronic ankle instability (CAI) affects postural control and balance, leading to alterations in center of pressure (COP) variables. Studies have shown that individuals with CAI exhibit decreased postural sway during single‐leg stance tasks [6] and altered neuromuscular control strategies during gait [7]. However, some research has found differences in COP variables between CAI and healthy individuals, such as lower correlation dimension of COP mediolateral displacement on stable surfaces [8]. Other studies have reported conflicting results such as Knapp et al. (2011) [9] found that no single force‐plate measure was highly effective in predicting CAI status, although some eyes‐closed, single‐limb measures showed limited predictive ability. These mixed findings highlight the complexity of CAI's impact on postural stability and the need for further research to identify the most sensitive COP variables for assessing balance deficits in individuals with CAI.

Building on previous research, which has demonstrated significant alterations in postural control among individuals with ankle injuries, this study aims to further investigate the effects of chronic ankle sprain (CAI) on center of pressure (COP) variables. Prior findings indicate that individuals with CAI exhibit greater COP velocity and excursion compared to both healthy controls and copers, while acute lateral ankle sprains (LAS) can lead to bilateral impairments in postural control [10, 11]. Despite these insights, it remains unclear whether an ankle injury affects COP‐related balance variables in the uninjured limb, highlighting the need for further investigation. Additionally, while previous studies have reported changes in COP range, variability, and moment arm [10, 12], the most sensitive COP variable to chronic ankle sprains has yet to be identified. Addressing these gaps, this study aims to determine whether an injury to one ankle induces changes in COP‐related balance parameters in the contralateral limb and which COP‐related variables are most affected by chronic ankle sprains. By elucidating these relationships, the findings of this study may contribute to improved assessments and rehabilitation strategies for individuals with recurrent ankle instability.

Given the established differences in postural control mechanisms between individuals with chronic ankle instability (CAI), lateral ankle sprain (LAS) copers, and healthy controls, this study seeks to refine current understanding of balance deficits associated with ankle sprains. Prior research has demonstrated that compensatory mechanisms, such as decreased postural sway in single‐leg stance, may emerge to mitigate the risk of recurrent sprains [13, 14, 15]. Given these biomechanical adaptations, it remains uncertain whether such changes extend to the uninjured limb, suggesting potential bilateral effects of unilateral injuries. Thus, we hypothesize that chronic ankle sprain will induce changes in COP‐related balance variables in the uninjured limb due to compensatory neuromuscular adjustments, and among the various COP parameters, the most sensitive indicator of impaired postural control in individuals with CAI will be identified. By determining the most sensitive COP measure to CAI, this study aims to refine clinical assessments and rehabilitation strategies, ultimately improving injury prevention and management for individuals with recurrent ankle instability.

## 2 Methods

### 2.1 Participants

A total of 40 participants were recruited, including 20 young adults with chronic ankle instability (CAI group) and 20 healthy individuals (control group). The sample size was determined using G*Power software (version 3.1.9.7) based on an effect size of 0.4, power of 0.80, and alpha level of 0.05 [16]. All participants in the CAI group were soccer players, referred from clinics in Hamadan to participate in the study.

The inclusion criteria for the CAI group were [1] a history of at least one significant ankle sprain [2], self‐reported sensations of “giving way” during activities [3], a Cumberland Ankle Instability Tool (CAIT) score of < 25 [17], and [4] no acute ankle injury symptoms. For the healthy group, the inclusion criteria were [1] no history of ankle sprain or lower limb injury [2], no balance or postural stability issues, and [3] no neurological or musculoskeletal disorders. The exclusion criteria for both groups included [1] a history of lower limb surgery or fracture [2], acute or chronic lower limb pain [3], neurological or vestibular disorders affecting balance, and [4] an inability to perform balance tests. All participants were informed about the study's purpose and procedures, and written consent was obtained. The study was approved by the Ethical Committee of the Islamic Azad University of Hamedan and adhered to the latest revision of the Declaration of Helsinki.

### 2.2 Instruments and Examination

The static balance of participants was assessed using a Kistler force plate (model 9286 BA, Kistler Instrument AG, Winterthur, Switzerland) with a sampling frequency of 1000 Hz. Data collection for COP movements commenced once the participant was positioned correctly on the force plate.

Static balance assessments included single‐leg stance tasks, where participants stood on either the left or right leg (or the injured and noninjured foot for the CAI group). Participants were instructed to maintain an upright posture in the center of the platform, remain as still as possible for 30 s, and avoid any unnecessary movements. The coordinates of the COP trajectory were recorded over time during each trial. All tests were conducted barefoot, with hands placed on the waist for consistency. The tests were performed under open‐eye conditions, with participants focusing on a fixed point at eye level on a wall 1.5 m away. During the single‐leg stance, the nonweight‐bearing leg was held at 90 degrees of knee flexion. Each balance task was repeated three times.

Data analysis was performed using Bioware software (version 3.5.2, Kistler Instrument AG, Winterthur, Switzerland). The raw data were filtered using a fourth‐order Butterworth filter with a cut‐off frequency of 20 Hz to remove noise and ensure accuracy [18].

The following traditional linear COP variables were analyzed based on previous research [8, 19]: COP Average Displacement (cm), which represents the mean length of the COP trajectory; COP SD Displacement (cm), reflecting the standard deviation of the COP trajectory length and indicating variability; RMS of COP, representing the root mean square of COP displacements to quantify the magnitude of oscillations; Range of COP, describing the total area covered by the COP movements; Slope of COP, indicating the rate of change in COP displacement over time; Acceleration of COP, which measures the rate of change in COP velocity; Velocity of COP, representing the speed of COP movements; and Integral of COP, reflecting the total cumulative displacement of the COP over time. All variables were evaluated in both the anterior‐posterior (AP) and medio‐lateral (ML) directions to provide a comprehensive analysis of postural stability.

### 2.3 Statistical Analyses

The Shapiro–Wilk test was used to assess the normality of data distributions and to confirm the suitability of parametric analysis. A three‐factor (group × foot × direction) mixed‐model repeated measures multivariate analysis of variance (MANOVA) was conducted to examine the effects of group (CAI vs. control), foot (injured vs. noninjured), and direction (anterior‐posterior [AP] vs. medio‐lateral [ML]) on multiple COP variables. When the multivariate test indicated significant differences, follow‐up univariate ANOVAs were performed to identify the specific dependent variables contributing to the effects. Partial eta squared (ηp^2^) was calculated to interpret the magnitude of main and interaction effects, with values of 0.01, 0.06, and 0.14 interpreted as small, medium, and large effects, respectively [20]. Although multiple comparisons were conducted, Bonferroni correction was not applied due to the exploratory nature of the study and the concern that overly conservative adjustments could obscure potentially meaningful findings. Instead, partial eta squared (ηp^2^) values were reported to aid in the interpretation of effect size and relevance. All analyses were conducted using SPSS version 26, with statistical significance set at *p* ≤ 0.05.

## 3 Results

**FIGURE 1:**
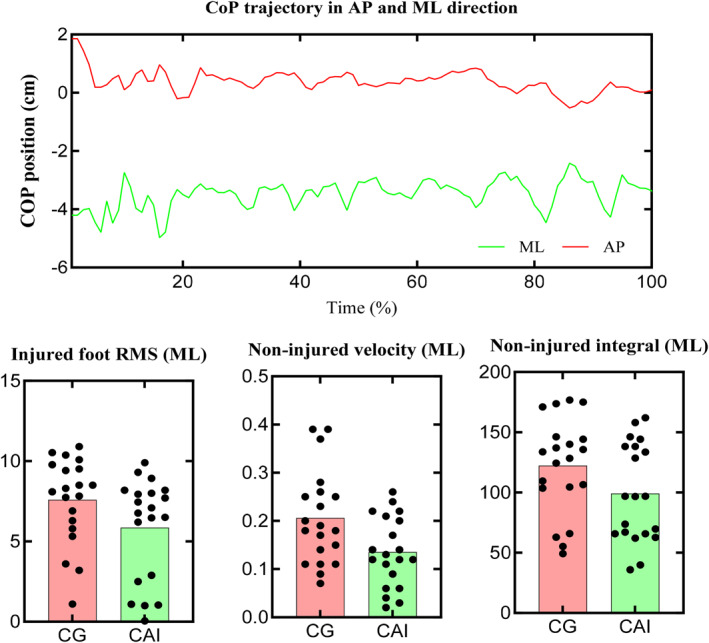
Top panel: Example of the CoP position signal (decomposed into AP and ML directions) for one participant. Bottom panel: Comparison between groups in RMS, velocity, and integral.

Comparative analysis of COP variables between the CAI and control groups for the injured limb demonstrated no statistically significant differences in the AP subset (Table 1). However, in the ML subset, RMS values were significantly elevated in the CAI group relative to controls (*p* = 0.049, ηp^2^ = 0.22) (Figure 1).

**TABLE 1:** Comparison of center of pressure (COP) variables between CAI and control groups for the injured limb.

	CAI	CG	F (*p*‐value)
Anrerior posterior			
Average	2.08 ± 0.41	2.32 ± 0.45	0.165 (0.690)
SD	0.95 ± 0.48	0.87 ± 0.30	0.173 (0.682)
RMS	2.33 ± 0.43	2.58 ± 0.41	0.189 (0.669)
Range	6.02 ± 1.62	4.71 ± 0.64	0.562 (0.463)
Slope	0.06 ± 0.01	0.04 ± 0.01	0.785 (0.395)
Acceleration	0.04 ± 0.01	0.04 ± 0.01	2.384 (0.140)
Velocity	0.51 ± 0.03	0.43 ± 0.02	2.874 (0.107)
Integral	41.68 ± 8.17	46.63 ± 9.08	0.164 (0.690)
Medial lateral			
Average	6.39 ± 0.85	5.29 ± 0.74	1.741 (0.204)
SD	0.83 ± 0.17	0.81 ± 0.09	0.013 (0.911)
RMS	7.48 ± 0.98	4.85 ± 0.74	4.689 (**0.049**)
Range	4.74 ± 0.98	3.85 ± 0.34	0.762 (0.394)
Slope	0.12 ± 0.06	0.02 ± 0.01	2.359 (0.142)
Acceleration	0.02 ± 0.01	0.01 ± 0.01	1.014 (0.327)
Velocity	0.19 ± 0.02	0.11 ± 0.03	1.518 (0.234)
Integral	155.95 ± 17.16	126.03 ± 14.97	1.744 (0.203)

*Note:* Values are presented as mean ± standard deviation. COP variables are reported in both the anterior‐posterior (AP) direction and the medial‐lateral (ML) direction. The measured variables include average (cm), standard deviation (SD, cm), root mean square (RMS, cm), range (cm), slope (unitless), acceleration (cm/s^2^), velocity (cm/s), and integral (cm^2^). The F‐statistic and *p*‐value are reported for comparisons between the CAI and CG groups. A *p*‐value < 0.05 indicates a statistically significant difference.

Abbreviations: CAI, Chronic Ankle Instability; CG, Control Group; COP, Center of Pressure; RMS, Root Mean Square; SD, Standard Deviation.

Regarding the noninjured limb, no statistically significant between‐group differences were detected in either AP or ML variables, except for ML velocity, which was significantly greater in the control group (*p* = 0.049, ηp^2^ = 0.21) (Table 2). Additionally, the integral value was significantly higher in the CAI group compared to controls (*p* = 0.042, ηp^2^ = 0.25) (Figure 1).

**TABLE 2:** Comparison of center of pressure (COP) variables between CAI and control groups for the noninjured limb.

	CAI	CG	F (*p*‐value)
Anrerior posterior			
Average	2.94 ± 0.54	2.03 ± 0.42	1.708 (0.208)
SD	0.93 ± 0.16	1.13 ± 0.17	0.765 (0.393)
RMS	3.15 ± 0.52	2.49 ± 0.35	1.094 (0.309)
Range	5.88 ± 1.55	6.63 ± 1.44	0.125 (0.728)
Slope	0.06 ± 0.01	0.10 ± 0.02	2.087 (0.166)
Acceleration	0.06 ± 0.01	0.05 ± 0.01	0.462 (0.506)
Velocity	0.66 ± 0.03	0.64 ± 0.04	0.184 (0.673)
Integral	58.80 ± 10.94	44.85 ± 7.74	1.082 (0.312)
Medial lateral			
Average	4.34 ± 0.81	7.02 ± 1.13	3.660 (0.072)
SD	0.99 ± 0.31	0.82 ± 0.09	0.254 (0.620)
RMS	4.74 ± 0.67	7.10 ± 1.11	3.207 (0.090)
Range	4.67 ± 1.17	4.22 ± 0.43	0.127 (0.725)
Slope	0.06 ± 0.02	0.06 ± 0.02	0.003 (0.958)
Acceleration	0.01 ± 0.01	0.01 ± 0.01	2.104 (0.164)
Velocity	0.12 ± 0.02	0.20 ± 0.03	4.126 (**0.049**)
Integral	140.03 ± 27.32	86.47 ± 21.55	4.659 (**0.042**)

*Note:* Values are presented as mean ± standard deviation. COP variables are reported in both the anterior‐posterior (AP) direction and the medial‐lateral (ML) direction. The measured variables include average (cm), standard deviation (SD, cm), root mean square (RMS, cm), range (cm), slope (unitless), acceleration (cm/s^2^), velocity (cm/s), and integral (cm^2^). The F‐statistic and *p*‐value are reported for comparisons between the CAI and CG groups. A *p*‐value < 0.05 indicates a statistically significant difference.

Abbreviations: CAI, Chronic Ankle Instability; CG, Control Group; COP, Center of Pressure; RMS, Root Mean Square; SD, Standard Deviation.

The results of the factor analysis are presented in Table 3. A significant main effect of direction was found for all COP parameters except for SD and slope (*p* < 0.05). The corresponding partial eta squared values indicated large effects for range (ηp^2^ = 0.35), acceleration and velocity (ηp^2^ = 0.95), as well as average, RMS, and integral (ηp^2^ = 0.70). Significant foot × direction interactions were observed for acceleration (*p* < 0.001, ηp^2^ = 0.57), velocity (*p* < 0.001, ηp^2^ = 0.65), and integral (*p* = 0.045, ηp^2^ = 0.22), all indicating large effects. The group effect was generally nonsignificant, except for RMS (*p* = 0.041, ηp^2^ = 0.22), which also represented a large effect, suggesting moderate group differences in postural stability. The CAI group consistently showed higher mean values. A significant main effect of foot was also found for acceleration (*p* = 0.011, ηp^2^ = 0.31) and velocity (*p* = 0.003, ηp^2^ = 0.40), both indicating large effects. Additionally, direction × group interactions were significant for COP average (*p* = 0.048, ηp^2^ = 0.19), RMS (*p* = 0.036, ηp^2^ = 0.23), acceleration (*p* = 0.026, ηp^2^ = 0.25), and velocity (*p* = 0.013, ηp^2^ = 0.30), all of which also fall within the large effect size category based on partial eta squared interpretation guidelines.

**TABLE 3:** Factor analysis of center of pressure (COP) variables in CAI and control groups.

	Group	Foot	Foot*group	Direction	Direction*group	Foot* direction
Average	2.40 (0.139)	1.70 (0.208)	0.000 (0.992)	45.58 (**0.000**)	4.12 (**0.048**)	3.83 (0.066)
SD	0.004 (0.947)	1.30 (0.268)	0.145 (0.708)	2.29 (0.147)	1.379 (0.256)	0.123 (0.730)
RMS	4.384 (**0.041**)	0.277 (0.605)	0.459 (0.507)	42.06 (**0.000**)	5.149 (**0.036**)	1.743 (0.203)
Range	0.129 (0.724)	0.632 (0.437)	0.949 (0.343)	9.942 (**0.006**)	0.194 (0.665)	1.01 (0.329)
Slope	0.743 (0.400)	0.20 (0.660)	2.903 (0.106)	0.006 (0.941)	2.434 (0.136)	1.107 (0.307)
Acceleration	0.007 (0.934)	7.979 (**0.011**)	0.319 (0.579)	319.38 (**0.000**)	5.863 (**0.026**)	24.39 (**0.000**)
Velocity	0.733 (0.120)	12.192 (**0.003**)	0.985 (0.334)	336.65 (**0.000**)	7.617 (**0.013**)	33.365 (**0.000**)
Integral	2.977 (0.102)	1.35 (0.261)	0.017 (0.896)	42.365 (**0.000**)	3.597 (0.074)	4.305 (**0.045**)

*Note:* Values in bold indicate statistically significant results (*p* < 0.05).

## 4 Discussion

The present study aimed to identify the most sensitive COP variables for assessing postural stability in individuals with CAI compared to healthy controls. The findings revealed nuanced differences in COP movement between the two groups, particularly in the mediolateral (ML) direction, and provided insights into the impact of CAI on postural control. These results have important implications for balance assessment and rehabilitation strategies, as well as for understanding the underlying mechanisms of postural instability in individuals with CAI. The most notable finding for the injured limb was the significantly elevated RMS values in the ML direction in the CAI group compared to controls. This suggests that individuals with CAI exhibit greater ML postural sway, likely due to impaired proprioceptive feedback and neuromuscular control in the injured ankle. The absence of significant differences in the AP direction aligns with previous studies indicating that ML stability is more affected than AP stability in CAI populations [21]. The elevated ML RMS values may reflect compensatory strategies to maintain balance, such as increased reliance on the hip strategy, which is often observed in individuals with CAI [10].

For the noninjured limb, the control group demonstrated significantly greater ML velocity (by 6%) compared to the CAI group, while the CAI group exhibited a notably higher integral value (by 62%). This substantial increase in integral COP values may reflect compensatory adaptations, wherein individuals with CAI unconsciously shift postural control strategies to rely more heavily on the noninjured limb due to impaired proprioception and neuromuscular control in the injured limb. A systematic review has confirmed proprioceptive deficits in CAI, including impaired joint position sense and kinesthesia, particularly when compared to the contralateral side [22]. Additionally, altered postural strategies involving the uninjured limb have been reported during gait initiation tasks in individuals with CAI. Such compensatory behaviors are consistent with findings in the neuromuscular control literature, suggesting an adaptive redistribution of postural demands to maintain balance [23, 24, 25]. Laterality bias, reflecting preferential reliance on the dominant or uninjured limb, may also contribute to this phenomenon. The exact mechanisms underlying these bilateral effects remain unclear and warrant further investigation, including assessments of proprioceptive and neuromuscular function in both limbs. The reduced ML velocity in the CAI group may indicate a more cautious or rigid postural strategy, possibly due to fear of re‐injury or altered neuromuscular control [26, 27]. Conversely, the elevated integral value in the CAI group suggests greater overall displacement of the COP, which may reflect less efficient postural control mechanisms. These findings highlight the bilateral nature of postural instability in CAI, as even the noninjured limb appears to be affected, albeit differently than the injured limb [28]. These findings align with recent research indicating that single‐limb stance tasks are sensitive enough to detect subtle postural control differences in both healthy and clinical populations [29]. Furthermore, evidence of strength deficits in the knee extensors and flexors of CAI patients suggests that neuromuscular impairments may extend beyond the ankle joint, potentially influencing overall postural strategies [30].

Factor analysis revealed a significant main effect of direction on most COP parameters, except for SD and slope. This underscores the importance of considering directional differences (AP vs. ML) when assessing postural stability, as the demands on the neuromuscular system vary between these planes. Significant foot*direction interactions were observed for acceleration, velocity, and integral, indicating that the influence of foot dominance or injury status on postural control is direction‐dependent. Additionally, significant direction*group interactions for COP average, RMS, acceleration, and velocity suggest that the CAI group exhibits distinct postural control patterns compared to controls, particularly in the ML direction. Although group effects were largely nonsignificant, the CAI group exhibited higher mean values for velocity and RMS, indicating subtle but meaningful differences in postural stability. These findings align with previous research demonstrating that individuals with CAI often exhibit increased postural sway and slower corrective responses [31]. The elevated velocity and RMS values may reflect a less stable postural system, which could predispose individuals with chronic AS to recurrent injuries and functional limitations [32].

The results of this study highlight the importance of assessing ML postural control in individuals with CAI, as this direction appears to be more sensitive to the effects of ankle instability. Clinicians should consider incorporating ML‐specific balance exercises into rehabilitation programs to address the identified deficits. Additionally, the bilateral differences observed in this study suggest that rehabilitation should not focus solely on the injured limb. Addressing deficits in the noninjured limb, such as reduced ML velocity, may help restore symmetry and improve overall postural stability [33, 34]. The findings of this study are consistent with previous research demonstrating that individuals with CAI exhibit increased postural sway, particularly in the ML direction [12, 35, 36]. However, the lack of significant differences in the AP direction contrasts with some studies that have reported AP instability in CAI populations [37]. This discrepancy may be due to differences in study protocols, such as the duration of balance tasks or the inclusion criteria for participants. The elevated integral value in the noninjured limb of the CAI group is a novel finding that warrants further investigation, as it suggests that CAI may have systemic effects on postural control.

The primary objective of this study was to identify the most sensitive COP variables for detecting postural stability deficits in individuals with CAI. Based on the results, the RMS in the ML direction emerged as a particularly sensitive measure, showing significant elevation in the CAI group compared to healthy controls. This finding aligns with previous research, such as Hertel and Corbett (2019), who also reported that ML postural sway is more affected than AP sway in individuals with CAI [21]. Additionally, the integral value, which reflects the overall displacement of the COP, was significantly higher in the CAI group for the noninjured limb, suggesting that this variable may also serve as a sensitive indicator of compensatory postural strategies. These findings are consistent with Wikstrom et al. (2010), who highlighted the role of ML stability in postural control deficits associated with CAI [10]. The elevated RMS and integral values in the CAI group likely reflect impaired proprioceptive feedback and neuromuscular control, which are common in CAI populations.

The results of our study highlighted RMS and integral values in the ML direction as the most sensitive COP parameters differentiating CAI from control participants. This aligns with prior research indicating that COP‐RMS is a robust and sensitive indicator of postural sway, especially under conditions requiring lateral stability. For instance, Neville et al. (2015) describe COP‐RMS as a representative measure of postural sway variance and note its utility in detecting balance impairments during single‐leg stance [38]. Moreover, Palmieri et al. (2002) recommend variables such as mean COP, RMS amplitude, and velocity to detect subtle balance changes in both AP and ML directions [39]. Integral values, reflecting cumulative displacement over time, similarly capture prolonged sway patterns and sensorimotor control deficits. Although no universal clinical cutoffs currently exist for these COP measures, their consistent elevation in ML sway among CAI participants—reflected in large effect sizes—supports their relevance. These findings underscore the importance of selecting COP variables that are sensitive to ML postural instability in CAI assessment. These results underscore the importance of incorporating ML‐specific COP variables, such as RMS and integral, into balance assessments and rehabilitation protocols to better capture postural stability deficits and guide targeted interventions. Future research should further validate these findings and explore their clinical utility in improving outcomes for individuals with chronic ankle instability.

This study had some limitations. Firstly, the relatively small sample size (*n* = 40) could have limited the statistical power and generalizability of the findings. Secondly, participants with CAI were exclusively male soccer players, which restricts external validity and the applicability of results to other populations, including females, athletes from other sports, and nonathletic individuals. Future studies should include larger, more diverse samples, with stratification based on sex, age, and different activity levels to enhance generalizability. Thirdly, the study employed a static single‐leg stance task with eyes open, which, while allowing precise measurement of COP parameters under controlled conditions, may not fully capture postural control deficits that emerge during more dynamic or functional activities. Clinically, balance impairments related to CAI often manifest under challenging conditions such as unstable surfaces, eyes closed, or during sport‐specific movements. Future studies are therefore encouraged to incorporate dynamic, perturbed, or dual‐task paradigms to increase ecological validity and enhance the clinical relevance of findings. Additionally, this study employed a cross‐sectional design without any clinical follow‐up, which limits the ability to determine whether the identified COP variables—such as ML RMS and integral—can predict functional performance or injury recurrence in individuals with CAI. Future longitudinal studies are needed to evaluate the predictive value and clinical relevance of these variables over time.

## 5 Conclusion

This study identified ML RMS as a sensitive COP variable for assessing postural stability in individuals with CAI. The findings underscore the importance of considering directional and bilateral differences in postural control, as individuals with chronic CAI exhibited elevated ML RMS and integral values, particularly in the injured limb. These subtle but meaningful variations in COP dynamics suggest impaired proprioceptive feedback and neuromuscular control, which may contribute to functional limitations and recurrent injuries. The results emphasize the need for targeted rehabilitation strategies that address ML‐specific postural control deficits, not only in the injured limb but also in the noninjured limb, to restore symmetry and improve overall balance. Future research should further validate these findings and explore the clinical utility of ML‐specific COP variables in improving outcomes for individuals with CAI. Additionally, longitudinal studies are needed to evaluate the efficacy of interventions aimed at reducing postural sway and enhancing neuromuscular control in this population. Although no universally accepted clinical thresholds currently exist for COP parameters such as RMS and integral, the current findings support their potential as sensitive markers of ML postural impairment. Establishing normative benchmarks through future studies may enhance their diagnostic and prognostic value in clinical settings.

## Author Contributions

**Mahdi Majlesi:** conceptualization, data curation, formal analysis, investigation, methodology, validation, visualization, writing – original draft, writing – review and editing.

## Conflicts of Interest

The author declares no conflicts of interest.

## Data Availability

The datasets generated and analyzed during the current study are available from the corresponding author on reasonable request.
